# Improving Patient Involvement in the Lifecycle of Medicines: Insights From the EUPATI BE Survey

**DOI:** 10.3389/fmed.2020.00036

**Published:** 2020-02-13

**Authors:** Lynda Grine, Rosanne Janssens, Eline van Overbeeke, Danielle Derijcke, Mitchell Silva, Belinda Delys, Isabelle Dusart, Veerle Aertsen, Magali Mertens de Wilmars, Joanna Robaczewska, Hilde Stevens

**Affiliations:** ^1^Department of Head & Skin, Ghent University, Ghent, Belgium; ^2^EUPATI BE VZW, Brussels, Belgium; ^3^Department of Pharmaceutical and Pharmacological Sciences, University of Leuven, Leuven, Belgium; ^4^The Synergist.org, Brussels, Belgium; ^5^Oncology, Novartis, Vilvoorde, Belgium; ^6^Vaccines, GlaxoSmithKline, Wavre, Belgium; ^7^Patient Expert, Innovative Medicines Initiative, Brussels, Belgium; ^8^Patient Expert, Travail & Cancer, Brussels, Belgium; ^9^Institute for Interdisciplinary Innovation in healthcare (I3h), Université Libre de Bruxelles, Brussels, Belgium

**Keywords:** patient involvement, patient engagement, survey, multi-stakeholder, medical innovation

## Abstract

EUPATI Belgium (EUPATI.be) is an informal gathering of local partners who are interested in improving patient involvement in healthcare innovation and medicines research and development. EUPATI.be brings together various stakeholders from different areas related to healthcare including patients, academia and industry. In doing so, we create an innovative collaborative approach where actors from different backgrounds work toward improving patient involvement in medical research, and putting the patient at the center of the Belgian healthcare system. Previously, we performed in-depth interviews with a small group of stakeholders on patient involvement. Here, we elaborate on our previous findings by using a nation-wide survey to inquire into Belgian stakeholders' perception on patient involvement. To this end, an electronic survey was available in French, Dutch and English, and accessible for 11 months. Twelve questions were asked, including 11 multiple choice questions and 1 open question. The latter was thematically analyzed according to the framework method. A total of 117 responses were registered and descriptive statistics were performed. The majority of respondents could be categorized into patient, academia and industry, whereas policy makers, payers, and healthcare professionals were underrepresented. We identified several barriers that hamper patient involvement, which were sometimes more reported by specific stakeholder groups. Next, we found that various stakeholders still consider patient involvement as a passive role, i.e., medical subject in a clinical trial. Respondents also reported that the role of the various stakeholders needed more clarification; this was also confirmed by the level of trust amongst the various stakeholders. Existing and the wish for more collaboration with the various stakeholders was reported by almost all respondents. Based on this survey, we can define the potential of involving patients in the medical research and development in the Belgian landscape. Our results will help to understand and tackle the various barriers that currently hamper patient involvement, whilst highlighting the need for a collaborative landscape from the multi-stakeholder perspective.

## Introduction

Patients want access to affordable healthcare and healthcare innovation. They are committed to invest and interact with healthcare professionals and researchers, the relevant authorities, and industry to speed up the medicines research and development (R&D) process. In addition, patients wish to provide their perspective and priorities to accelerate the development of precision medicine, targeted therapies and personalized care. Patient involvement is increasingly recognized as an integral part of healthcare and a critical component of safe and effective people-centered services. Engaged patients are more able to make informed decisions about their care options (1). Furthermore, resources may be better used if they are aligned with patients' priorities and this is critical for the sustainability of healthcare systems worldwide. Moreover, patient involvement also benefits public support for biomedical research. A transparent, timely and structured dialogue between all stakeholders in the healthcare sector is the key ingredient to reach those goals, which we confirmed while surveying the lifecycle of medicines (2).

Patient-centered initiatives, such as the Innovative Medicines Initiative (IMI) have greatly added to the awareness that patients need to be at the center of a healthcare system. In order to participate in this innovation process, the European Patients' Academy on Therapeutic Innovation (EUPATI), a patient-led public-private partnership was created in 2012 (1). EUPATI aims to increase patient involvement by developing educational toolboxes and trainings. Since communication needs to occur in the patient's mother tongue with patients has to happen in the patient's own language, EUPATI has facilitated the creation of national platforms (ENPs). EUPATI.be, a platform for patient education established as the Belgian chapter of EUPATI, has been created in 2017. EUPATI.be's main goal is to empower patients through education. Led by patients, EUPATI.be provides trainings based on the EUPATI toolboxes to render patients, and other healthcare stakeholders, more educated about the role they can play in the drug research and development process. Furthermore, EUPATI.be aims to foster the national debate and wants to be a key partner for government when developing relevant health research legislation, policy and practice (2). By broadly promoting patient involvement, patients become active partners in medicine development.

While Belgium has already been experiencing a shift from “reactive care” to P4 medicine emphasizing predictive, preventive, personalized, and participatory care, the patient's role in this process is still not well-understood and needs to be better defined (3). Better understanding of key drivers, barriers and mechanism for effectively engaging the patient in medical research is essential to improve the patient's experience and optimize healthcare systems (2). Our study provides an important contribution in this direction by looking into the perception of patient involvement in medical research from the perspective of various stakeholders, including patients, healthcare professionals, industry and policy makers. Bringing this multi-stakeholder perspective on patient involvement is crucial for developing mutual understanding and further strengthening the patient's roles in healthcare innovation.

## Methods

### Survey Design

The survey was designed by the EUPATI.be Executive Committee (ExCom), a balanced group of academics, patient and industry representatives, to gain insight in the landscape of stakeholders in patient involvement in Belgium, with a total of 12 questions included (Supplementary File). Questions were included on the following topics: patient role in medical research, collaboration, understanding, trust, and barriers. Respondents were asked to score statements for importance/frequency, with one as not important at all/never; two as slightly important/rarely; three as moderately important/sometimes; four as very important/often; and five as extremely important/very often. An open-ended question was included to elaborate on specific topics. The survey was developed in three languages: Dutch, French, and English. Survey questions were checked for clarity and comprehensibility by various stakeholders attending a EUPATI.be Workshop in October 2017; no adaptations were deemed necessary. SurveyMonkey was used for the survey and to collect data. Dissemination of the survey was done through the website of EUPATI.be, and social media, including social media accounts of individual EUPATI.be ExCom members, and their networks. In addition, the survey was sent by email to affiliated research departments of academic Executive Committee members. The survey was accessible online from November 2017 until October 2018. Since this was a non-experimental, voluntary survey, where no personal and sensitive data was collected, no ethical approval was obtained.

### Survey Analysis

#### Multiple Choice Questions

Responses were coded for analysis and descriptive statistics were performed per question. All participants were included, regardless of whether they completed the survey. In case interpretations required the role of the respondent (e.g., patient or industry), only the answers from respondents who reported their roles were included. In case similar response rates were observed across comparable categories, answers were pooled: e.g., an encompassing category “Advising other stakeholders” was created to pool the following answers “Advising pharmaceutical companies,” “Advising academic researchers,” “Advising regulatory agencies,” “Advising healthcare professionals,” “Advising HTA agencies,” “Advising reimbursement agencies,” and “Advising policy makers.”

Statistics and graphs were performed and created with GraphPad Prism (version 7, San Diego, USA).

#### Open-Ended Question

The open-ended question asking for participants' opinions on how to improve patient involvement was analyzed thematically using the framework method as described by Lacey and Luff (3). Table 1 explains the implementation of the five iterative stages of the framework method: (1) familiarization, (2) identifying a thematic framework, (3) coding, (4) charting, and (5) mapping and interpretation. The triangulation of the involved researchers is shown in Table 1 as well.

**Table 1:** Stages of framework method.

1. Familiarization	The answers to the open question 4 of the survey were thoroughly read by the researchers involved in the analysis (RJ, DD)
2. Identifying a thematic framework	RJ and DD independently assigned a label to all the answers (“open coding”) before meeting to develop the initial list of codes, i.e., the initial coding framework. The initial framework was further discussed, refined and agreed upon among the researchers involved in the analysis (LL, EO)
3. Coding	To assign the codes from the framework, the answers were color-coded by one researcher (RJ), meaning that each answer was given a color matching the code it belonged to
4. Charting	Excel was used to create a framework matrix for each code from the final framework by one researcher (RJ)
5. Mapping and interpretation	Using the Excel framework matrix created in stage 4, RJ and DD independently searched for themes in the data and convened afterwards to discuss their interpretations (investigators' triangulation). During these discussions, RJ and DD reached consensus about stakeholders' proposed solutions on how to improve patient involvement and subsequently derived the themes and illustrative quotes in the results sections More Education and Information, More Favorable Regulatory and Ethics Environment, More Awareness, Communication and Trust, and A Systematic and Structured Approach. This stage was guided by the research question, the barriers for patient involvement highlighted by participants in question 3 and a careful analysis of what was in the data. Interpretations were made by reviewing the matrix and making associations within codes and interviewees, as well as between codes and interviewees. Whenever the data was rich enough, the interpretations generated in this stage went beyond the description of a particular interviewee to the explanation of potential reasons or beliefs of multiple interviewees

## Results

### Participants' Demographics

**Figure 1:**
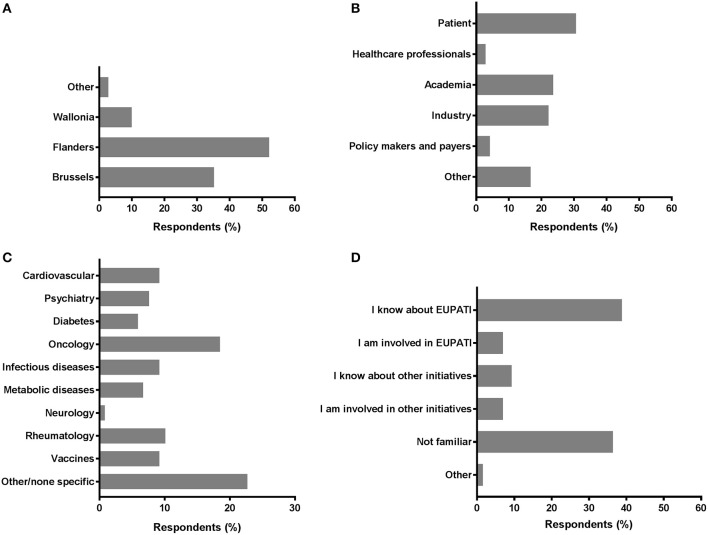
Characteristics of survey respondents. Respondents were inquired into a selection of basic demographics such as **(A)** region; **(B)** role; **(C)** disease field; and **(D)** familiarity with EUPATI (-like) initiatives.

A total of 117 individual respondents participated, but not all respondents completed the full survey. The demographics of the respondents is presented in Figure 1. The majority of respondents were located in Flanders (52.1%) and in Brussels (35.2%). Wallonia and other regions were less represented; 9.9 and 2.8%, respectively (Figure 1A). We observed comparable contributions from the patient community (30.6%; including individual patients and (non)-patient representatives), academia (23.6%), and the industry (22.2%) (Figure 1B). Less than one out of five was represented by “Other.” Policy makers, payers and healthcare professionals were the least represented among respondents (4.2 and 2.8%, respectively). The therapeutic areas were variably represented (Figure 1C); the majority of respondents were related to areas not predefined in the survey (22.7%) and oncology was the second-most represented (18.5%). Representation from the fields of rheumatology, vaccines, cardiovascular, and infectious diseases were also considerably present (9.2–10.1%). More than a third was familiar with EUPATI (38.8%), whereas a comparable portion (36.4%) of respondents were unfamiliar with EUPATI or other initiatives and projects on patient involvement and education in healthcare innovation. Still, more than 1 out of 10 either knew about or were involved in other initiatives (9.3 and 7.0%, respectively; Figure 1D).

### Patient Involvement: Settings and Importance

**Figure 2:**
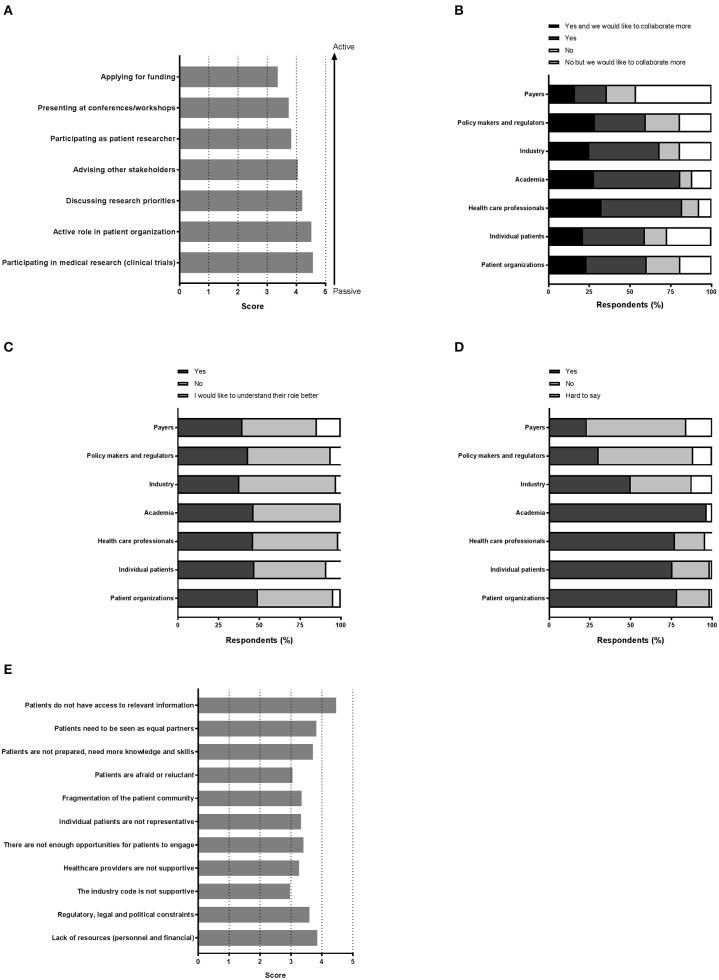
“Perception on the various aspects of patient involvement.” Respondents were asked to reply to statements with a degree of agreement on aspects including **(A)** the importance of the patient in medical research, **(B)** collaboration, **(C)** the role of each stakeholder, **(D)** trust, and finally **(E)** barriers.

Various settings were presented in which patients could be involved and participants were asked to indicate how important patient involvement would be in each setting. The results are illustrated in Figure 2A. Although no setting scored below 3, a subtle ranking could be observed in the average importance rated by respondents. To highlight these subtle differences, a gradient of passive to active participation is shown on the right in Figure 2A. Overall, participation in medical research as subjects was found as the most important setting for patient involvement, whereas application for funding was considered least important. A subanalysis per stakeholder was made to characterize the importance in-depth and is presented in Table 2. Here, we found that the industrial stakeholders perceive participation as medical subjects as the most important form of patient involvement. The subanalysis showed no remarkable deviations in average scores although policy makers and payer representatives found “presenting at conferences and workshops” less important than other stakeholders. Furthermore, the same group also found participation in application for funding together with academia and industry less important, with an average score of 2.3, whereas other scores ranged from 3.3 to 4.1 amongst the rest. Remarkably, active participation in patient organizations was highly rated by all stakeholder communities.

**Table 2:** Patient involvement rated per stakeholder.

	**Patient community**	**Hcp and academia**	**Industry community**	**Policy and payers**	**Other**
Applying for funding	4.0	3.3	3.4	2.3	4.1
Presenting at conferences/workshops	4.1	3.7	4.0	2.8	4.5
Participating as patient researcher	4.4	3.8	3.9	3.5	4.2
Advising other stakeholders	4.6	3.9	4.4	3.6	4.5
Discussing research priorities	4.4	3.8	4.3	3.8	4.7
Active role in patient organizations	4.9	4.4	4.5	4.8	4.6
Participating in medical research (clinical trials)	4.5	4.5	4.9	4.5	4.7

### Collaboration With Actors in Healthcare Innovation

Figure 2B illustrates the collaboration with the various stakeholders for healthcare innovation. Respondents could also indicate whether more collaboration was preferred. With almost half of the votes, payers were not involved in healthcare innovation, although 17.9% of the respondents reported a wish to collaborate more with this stakeholder group. Not surprisingly, academia and healthcare professionals represented the major collaborators, whereas the latter were also seen as potential added value for future collaborations (32.8% to collaborate more). Patient organizations and individual patients each represented more than a third with whom collaborations are ongoing, and up to 23.5% wanted more collaborations with these actors.

### Understanding Actor's Roles in Medical Research and Development

Respondents were also asked if they knew the role of each actor in medical research and development (Figure 2C). Very similar numbers were found for each actor and for each category of response, except for the payers' role which was perceived as least understood (14.7%). The roles were however not entirely clear, since approximately half of respondents wished to better understand all roles, with percentages ranging from 44.1 to 59.4%, indicative for a gap in healthcare innovation.

### Trust in the Different Stakeholders

Next, we sought to understand the trust amongst stakeholders, which is illustrated in Figure 2D. Surprisingly, trust in academia surpassed the trust instilled in patient organizations or individual patients (96.67 vs. 76.57, and 75.71%, respectively). Healthcare professionals were trusted by a comparable number of respondents (77.14%). Yet, for these actors, about 1 out of 4 respondents indicated to not trust them. Even more surprising was the low trust found in the groups policy makers, regulators and payers, reaching only a maximum of 30.42%. A minority reported to have no trust in these actors; the remaining found it hard to say. The industry was trusted by 50% of the respondents; whilst 37.50% indicated to find it hard to say whether they trusted the industry.

### Patient Involvement: Barriers and Solutions

Among 73 participants who answered the open-ended question, 16 were academics, 4 healthcare professionals, 20 patient organizations, 31 industry, and 2 policy-makers/regulators. The barriers and solutions for patient involvement indicated by participants related to (i) education and information, (ii) regulatory and ethics environment, (iii) awareness, communication and trust, and (iv) systematic and structured approach. None of the presented barriers in the survey (Figure 2E) received an average score below 3.0, indicating that the average frequency with which participants encountered these barriers ranged from sometimes (3) to very often (5). The three barriers receiving the highest average scores were: “*patients do not have access to relevant information”* (4.5), “*lack of financial resources and personnel”* (3.9) and “*patients need to be seen as equal partners”* (3.8). Conversely, “*the industry code is not supportive”* (3.0), “*patients are reluctant or afraid”* (3.0) and “*healthcare providers are not supportive”* (3.3) were the three barriers receiving the lowest average scores. Two barriers were identified as often hampering including patients, including “*the lack of acknowledging the patient as a partner*” and “*the lack of knowledge and skills amongst patients*.” Practical issues such as regulatory, legal and political constraints and lack of resources such as personnel and finances were also indicated as “often.” All other barriers were still present as none scored 'never frequented'. When analyzing the barriers in detail per stakeholder (Table 3), we observed that financial resources were reported as a major barrier by most stakeholders: the highest and lowest frequency in the “other stakeholders” and “policy and payer” categories, respectively. Similarly, lack of resources such as finances and personnel was also seen as a significant barrier; where the lowest score was again in the policy and payer category. The same stakeholder group also scored least for the barrier of “fear amongst patients to participate in medical research due to risks.” This stakeholder, however, rated a fragmented patient community as a major barrier, which was also rated highly amongst the other stakeholders. Interestingly, the industrial community also indicated this as their top barrier. Furthermore, the latter also viewed the lack of involvement between patients and healthcare professionals due to lack of knowledge and skills on the patients' side as a barrier. The solutions described by participants are detailed below and in Table 4.

**Table 3:** Barriers encountered per stakeholder.

	**Patient community**	**Hcp and academia**	**Industry community**	**Policy and payers**	**Other**
Patients do not have access to relevant information	3.4	3.1	3.8	2.5	4.3
Patients need to be seen as equal partners	4.5	3.4	3.6	4.0	4.3
Patients are not prepared, need more knowledge and skills	4.0	3.4	3.5	3.5	4.3
Patients are afraid or reluctant	3.3	3.0	3.2	2.5	3.5
Fragmentation of the patient community	3.6	3.4	3.6	3.9	4.0
Individual patients are not representative	3.5	2.9	3.1	2.5	3.3
There are not enough opportunities for patients to engage	4.0	2.3	3.9	3.8	3.8
Healthcare providers are not supportive	3.2	4.2	3.3	2.8	3.8
The industry code is not supportive	2.8	3.0	3.7	2.5	3.4
Regulatory, legal and political constraints	3.8	3.5	4.0	2.0	4.2
Lack of resources (personnel and financial)	4.6	4.3	3.8	2.8	4.2

**Table 4:** Barriers and solutions proposed by the respondents.

	**Barrier: a lack of**	**Solution: need for more**
Education and information	•Patients are not prepared and need more knowledge and skills •Patients do not have access to relevant information	•About drug development & disease process •To increase patients' confidence & trust regarding drug development •Using interactive approaches •Via patient organizations, schools & healthcare professionals
Favorable regulatory and ethics environment	•Regulatory, legal, and political constraints •Industry code is not supportive •There are not enough opportunities for patients to engage	•To create a bigger incentive •Toward pharmaceutical companies and healthcare professionals •Via a clear position statement from the government and clear guidelines on how to approach patients
Awareness, communication, and trust	•Patients are reluctant or afraid •Patients need to be seen as equal partners •Healthcare professionals are not supportive	•Amongst healthcare stakeholders •Align stakeholders on the role of patients in R&D •Conferences, meetings and awareness campaigns
Systematic and structured approach	•Lack of financial resources and personnel •Fragmentation of the patient community •Individual patients are not representative	•Involve patients early and at all phases of drug R&D •Ensure a representative voice of patients when participating in discussions •Recognize and compensate patients for their contributions •Facilitate information sharing & collaboration between stakeholders

#### More Education and Information

Participants from different stakeholder groups (academics, patient organizations, patients, industry, policy makers) described that in order to be able to participate in discussions on topics about drug research and development (e.g., clinical trial design), patients should be better informed and educated about their disease and how drug development works:

“*First we have to make sure patients can easily access the information necessary to be able to speak about a certain topic. Then we could have them sit at the table as a full-fledged partner in the debate.” (patient organization representative)*

Some participants mentioned that not only patients, but also other healthcare stakeholders, such as patient organizations, the general public, and journalists need to be better informed about the drug development process. Participants highlighted that more information is needed about how clinical trials work and that this information should be adapted to patient needs and be clear, simple, interactive but also honest, up-to-date and correct. Access to such information would also increase patients' confidence to participate in discussions and their trust in pharmaceutical companies and drug development according to two participants (see below). Specific approaches for informing and educating patients brought forward were via: (i) media (“digitization”), e.g., infographics or videos, (ii) interactive conversations in the medical doctors' office to receive patients' feedback, (iii) workshops in which patients are informed and educated by researchers and physicians on their disease, and (iv) brochures developed by the government. Several patient organization representatives mentioned that *specific* education should be available to train patients as “patient experts.” One respondent further explained that such training should include a quality label, and another respondent proposed that such experts should be present in all pathologies. Some respondents suggested channels to disseminate information, including (i) schools (to educate the general public on drug development), (ii) patient organizations, and (iii) healthcare professionals, including general practitioners and specialists.

#### More Favorable Regulatory and Ethics Environment

Some participants described how patient involvement could be achieved by providing a bigger regulatory and ethical incentive toward pharmaceutical companies and healthcare providers. At the regulatory level, some participants argued that the government^1^ and politicians need to have an unambiguous opinion toward stakeholders on the desirability of patient involvement and push patient involvement toward healthcare providers and industry. Some continued that patient involvement and education should be made mandatory:

“*A bigger policy push forcing industry and healthcare providers to include patients” (industry/ professional association member)*

In this context, one industry member specifically mentioned that more interaction between politics and industry is needed. Another industry member was convinced that the involvement of patients by industry is currently ethically prohibited because of “*bias risk,”* and a third industry member pointed out that clear guidelines on how industry can approach patients should be made available.

#### More Awareness, Communication, and Trust

Patients have a pivotal role to play in medical R&D and clarifying this role and associated responsibilities was expressed as an opportunity to align different partners to obtain more impactful patient involvement. In order to respect patients as equal partners, their role must be well-understood by all players. To this end, we identified the need to improve the general awareness about patient involvement. This could be obtained through different means, including events such as awareness campaigns, patient involvement conferences, EUPATI-events and/or patient organization meetings. The latter would allow early and timely detection of patient's needs and priorities and enable early patient involvement.

“*We need to inform the general public on what is clinical research. Still too many patients do not believe in research or trust companies” (academic rep)*

Furthermore, patients want to be kept informed about the latest research updates and learn about patient involvement opportunities in different areas and with different stakeholders. A few examples included regular meetings with pharma.be, the federal agency for medicines and health products (FAMHP), researchers, and hospitals. In addition, by providing more information, for instance on clinical studies, may directly address the patients' fears related to medical R&D, hereby improving dialogue with and trust toward different stakeholders.

#### A Systematic and Structured Approach

Involving patients early during medical R&D was acknowledged as a crucial factor. This also included the involvement of patients at every level of the hospital-management pyramid. Allowing patients to co-design the endpoints of a clinical study and study protocols overall was described as one of the key activities where patients can add a lot of value. Moreover, communication between patient and physician was also reported: 3 different stakeholders (patient, doctor, and academic) specifically addressed the need for more time for dialogue during consultations.

“*Patient empowerment should be enhanced through a carefully designed healthcare team that regularly engages with the patient to identify needs”*

In addition to a better understanding of the roles, representation of stakeholders was identified as a quality criterion to a multi-stakeholder approach. Remarkably, one respondent thought that a patient is unable to represent a broader patient community. This was reinforced by the notion that the role of the patient is not sufficiently valued: fair compensation and recognition for patients and patient representatives was identified as a valuable opportunity. Some solutions included considering subsidized patient advocate roles and compensating patient organizations. This in itself would tackle a financial issue since patient organizations often struggle with funding. It was also postulated that companies that truly invest in patient involvement could be rewarded, hereby stimulating the industry. The latter two arguments would simultaneously address the bias risk mentioned previously in section c.

According to the respondents, the availability and accessibility of accurate information is still challenging. Therefore, easy access to information in an open infrastructure, accessible to different types of stakeholders may facilitate interactions and collaboration. Guidance and tools to support a broad range of patient representatives are needed to enable them, and may also be beneficial for other stakeholders.

## Discussion

This study sheds light on the Belgian landscape for patient involvement where multiple stakeholders in medical innovation report on their experiences. EUPATI.be's mission to facilitate development of patient expertise is highly dependent on its position in this landscape: this is reflected by the main finding that more than a third of the 117 respondents is familiar with EUPATI.be. Understanding how the different stakeholders perceive patient involvement is key to develop a platform to facilitate interaction between patients and stakeholders. Our data shows that patient involvement is still seen as the participation of patients as medical subjects in research, rather than an active and equal partner in decision making at the research, medical and regulatory level. This in itself needs to be addressed by creating an ecosystem in which patient expertise is acknowledged and valued by different stakeholders. However, this acknowledgement will only occur when patient input is provided professionally in a culture where patient (experts) are perceived as equal partners. Regulatory agencies, such as the European Medicines Agency (EMA), have led by example, involving patient organizations and their disease-specific expertise to revise the design of clinical trials, e.g., the Medicines Adaptive Pathways to Patients (MAPPS) (4).

EUPATI's guidelines on patient involvement distinguishes 4 domains, including pharmaceutical industry-led medicines R&D, ethics committees, regulatory processes, and health technology assessment (HTA). Although patient involvement in R&D may seem obvious, there remains a need for platforms: EUPATI and **IMI** both focus on facilitating this interaction with and for the industry. In addition, different initiatives exist such as the Patient-Centered Outcomes Research Institute (PCORI), FDA's Patient-Focused Drug Development (PFDD) initiative, Clinical Trials Transformation Initiative (CTTI), and the Patient Focused Medicine Development (PFMD) coalition. The involvement of patients during HTA seems less obvious, although this is where another major impact on patients occurs - between scientific evidence and decision making. The Belgian Health Technology Assessment “KCE” has published their position on patient involvement in November 2019, stating the needs for structural and organizational requirements and revisiting their internal culture on patient involvement, with a main focus on how patient involvement is to be regarded as complementary to scientific evidence (5). But also funding agencies are increasingly aware of the need for dialogue. In May 2019, the King Baudouin Foundation organized a symposium “Mind the Gap” on how different stakeholders can collaborate to set research priorities (6), exemplified by the UK-based James Lind Alliance (http://www.jla.nihr.ac.uk/). Indeed, our findings confirm that, despite many topdown and bottom-up efforts (7, 8), true patient involvement remains challenging.

Our survey generated an overview of the foremost barriers hampering patient involvement experienced by the different stakeholders, and simultaneously allowed respondents to suggest solutions. These suggested “bottom-up” approaches may form the most immediate solutions to tackle the challenges. The four barriers “lack of education and information,” “lack of favorable regulatory and ethics environment,” “lack of awareness, communication, and trust,” and “lack of a systematic and structured approach” are considered most urgent to be tackled. The high rate of clinical trial failures represents an ideal opportunity to explore these barriers and solutions during the different steps of clinical trials (9). However, patient involvement can also occur at a more practical level as evidenced by a case study on pharmaceutical packaging (10).

Furthermore, our study shows that, for the Belgian ecosystem, there is a clear willingness to increase patient involvement at various levels, despite existing barriers. EUPATI.be aims to remove the existing obstacles by filling the gaps defined in the ecosystem of patient involvement by focusing on patient education, awareness and strategic collaborations, forming the basis to expand the roadmap of EUPATI.be.

### Patient Education

Effective patient involvement is fundamental to boost the safety and efficacy of novel therapies, whilst ensuring that novel treatments truly cater to the patients' needs. To increase patient involvement in medical R&D process, patients need to be armed with a deep understanding of the healthcare ecosystem during the various steps in the development process, including terminology. Educating patients to become bespoke experts and advocates is therefore key (11). Therefore, EUPATI.be's mission is to provide this education: complementary to the EU EUPATI course of 270 h online training (based on the EUPATI toolboxes and face-to-face meetings), we also develop educational workshops for the broad public. These workshops are rather topic-specific than disease-specific, and its modules are developed to relate to general knowledge, such as clinical trials, biological drugs and vaccine confidence. The workshops can also be tailor-made for the fragmented and multi-faceted Belgian healthcare system. In fact, due to Belgium's federal and regional infrastructure, a myriad of organizations and institutions are active in the healthcare system, leading to a multitude of healthcare stakeholders involved to provide health services, in particular diagnosis, treatment, and cure, but also, and increasingly important, prevention to the patient. Although other actors, such as agencies for pricing and reimbursement, HTA or insurance companies, do not directly provide care to the patient, they also play a crucial role in the accessibility of healthcare services. To deal with this complexity and improve understanding amongst the stakeholders, EUPATI.be's workshops explain the roles, tasks, the mission and the responsibilities of the various Belgian healthcare actors. The workshops are driven by demand and can be developed upon request. The ultimate aim of these interactive workshops is to create better awareness of how and where in the process patients can provide input and hence be the driving force in the clinical development of new therapies.

### Creating Awareness

A White Paper will be published on the current status and opportunities of patient involvement in Belgium based on the 2018 survey. This White Paper will be widely distributed to patient organizations, policy makers, academics, industry and clinicians. Furthermore, EUPATI.be will continue to carry out its mission on public events, including its own annual conference, whilst pro-actively contacting patient organizations to set up long-term collaborations.

### Strategic Collaborations

Collaboration is essential for innovation in healthcare and in order to make collaborations successful and sustainable, all partners should be considered as equals. Hitherto, trust is a key concept, and all actors need to align on a common mission and vision. Since strong collaborations often build upon bridging knowledge gaps and crossing disciplines, EUPATI.be will leverage its strength of being composed by members from patient organizations, academia, and industry to enhance awareness about patients' needs and preferences, and facilitate communication and trust between the various stakeholders. Hitherto, all stakeholders can plea with the governing authorities and regulatory bodies for an unambiguous position on how to increase patient involvement. EUPATI has guidelines that can be employed for this stance (e.g., the EUPATI Guidelines) (4, 12, 13). By collaborating strategically, EUPATI.be can establish a platform where patients and decision-makers can interact in a more structured and ethical way. This will require major efforts to establish trust between all involved stakeholders.

This report has several strengths and limitations. Not all respondents completed the survey in its entirety, introducing a potential bias in the preference to answer certain questions related to the respondent's background as a stakeholder. Due to anonymity and the modest sample size, generalization should be done with caution. Furthermore, The survey was open for 12 months, allowing as many respondents as possible to participate. However, the extended time frame may also have influenced the opinions. Furthermore, it was accessible online and the survey could be shared by non-EUPATI.be members enabling to reach a wider audience—no specific audience was targeted-, yet we only reached a modest sample size. It was a nation-wide bottom-up survey: respondents reflect the actual interest in patient involvement. However, this is a limitation as well since respondents interested in patient involvement may be more likely to participate to the survey—leading to a bias in the results, and uncertainty about actual representation of the stakeholders in the Belgian landscape. Data was collected anonymously, thus identities could not be verified, hampering interpretation of the answers' context. Furthermore, based on the demographics we observed an underrepresentation of several stakeholders, including policy and payer representatives. Although the intention was to perform a nation-wide survey, the majority of respondents were from Flanders or Brussels, complicating interpretation for Belgium in general. Yet, by not targeting a specific stakeholder, we provide survey-based data from a multi-stakeholder perspective to enable mutual understanding. This study can be viewed as a scientific milestone in understanding the multi-stakeholder perspective at the national level and can serve as a guidance for a pan-European study. Lastly, based on this survey, EUPATI.be can use evidence-based data to formulate priorities within its mission and anticipate country-specific obstacles in order to enable patient involvement.

## Conclusion

Patient involvement in the lifecycle of medicines has been recognized as one of the most promising approaches to promote innovation in healthcare. EUPATI.be represents a practical example of how patient involvement can be improved through patient education, creating awareness and establishing collaborations. EUPATI.be's strategic work in these areas is driven by the multi-stakeholder processes and the active involvement of patients, industry and academia is crucial for creating a patient-centric ecosystem—a new model helping patients to better engage in their role. Here, we report on the Belgian perception of patient involvement from different stakeholders' points of view. EUPATI.be's mission to facilitate patient participation remains largely unknown amongst respondents. Moreover, interpretation of patient involvement remains mainly as study subjects in medical research, indicative that a revision of the definition of “patient involvement” is primordial. Based on these results, we believe that EUPATI.be's mission must be reinterpreted in priority. To achieve this, we need policy makers to promote a regulatory environment and create a sustainable framework where patient involvement and its current barriers can be overcome, when funding medical research.

Ultimately, better coordination amongst various initiatives and levels of intervention will ensure long term improvement in patient involvement.

## Data Availability Statement

Datasets are available upon request addressed to the corresponding author at Lynda.Grine@ugent.be.

## Author Contributions

JR and HS supervised the project. All authors helped to develop the survey, checked for comprehensibility, released and promoted the survey through their networks, provided intellectual input for interpreting data, and approved the manuscript's content for publication. RJ and EO extracted and coded data. LG, RJ, and EO analyzed the data, and drafted the manuscript.

### Conflict of Interest

BD was employed by the company Novartis Pharma NV, DD was employed by the company The Synergist.org. ID was employed by the company GlaxoSmithKline NV. MM was employed by the company Travail & Cancer. The remaining authors declare that the research was conducted in the absence of any commercial or financial relationships that could be construed as a potential conflict of interest.

## Footnotes

^1^Both the government on the national level, the “Federal Agency for Medicines and Health Products” (FAMHP), and the government on the European level were mentioned.
