# Supplementary material for: Improving Patient Involvement in the Lifecycle of Medicines: Insights From the EUPATI BE Survey
Source: Front Med (Lausanne). 2020 Feb 13;7:36. doi: 10.3389/fmed.2020.00036 (PMC7031274; doi:10.3389/fmed.2020.00036)
Supplement: Supplementary file 1 [file Presentation_1.pdf]

Study on patient involvement and mapping the ecosystem of ENP Belgium.

Thank you for participating in the survey, which is part of the research conducted by EUPATI Belgium. The survey aims to identify the various challenges and barriers of patient involvement in Belgium, and the results will inform the actions of EUPATI Belgium.

The survey should take about 15 minutes to complete.

EUPATI Belgium is an informal gathering of local partners who are interested in improving patient involvement in healthcare innovation (medicines research and development, medical devices, vaccines,..). EUPATI Belgium bridges various stakeholders from different areas related to healthcare innovation with a focus on the patient.

To learn more about EUPATI Belgium, please visit http://www.eupati.be/.

If you have any questions regarding the study, please contact info@eupati.be.

1. ARE YOU FAMILIAR WITH EUPATI OR OTHER PROJECTS AND INITIATIVES TO SUPPORT PATIENT INVOLVEMENT IN MEDICAL RESEARCH AND DEVELOPMENT? SELECT ALL THAT APPLY AND PROVIDE EXAMPLES.

☐ I know about EUPATI

☐ I am involved in EUPATI as….

☐ I know about other initiatives such as……

☐ I am involved in other initiatives such as….

☐ NO

☐ Other, please specify:

2. IN YOUR OPINION HOW IMPORTANT IS TO INVOLVE PATIENTS IN THE FOLLOWING ROLES?

	Extremely important	Very important	Moderately important	Slightly important	Not important at all
Advising health care professionals	☐	☐	☐	☐	☐
Advising a pharamceutical company	☐	☐	☐	☐	☐
Advising a regulatory agency	☐	☐	☐	☐	☐
Advising a reimbursement agency	☐	☐	☐	☐	☐
Advising policy makers	☐	☐	☐	☐	☐
Advising academic researchers	☐	☐	☐	☐	☐
Presenting at conferences, workshops	☐	☐	☐	☐	☐
Taking an active role in patient organisations	☐	☐	☐	☐	☐
Participating in medical research, i.e. clinical trials	☐	☐	☐	☐	☐
Participating in public debates and discussions on research priorities	☐	☐	☐	☐	☐
Applying for grants and funding with industry and academia	☐	☐	☐	☐	☐
Participating in projects as a patient researcher who for example undertakes interviews with study participants	☐	☐	☐	☐	☐

Other, please specify:

3. HOW OFTEN DO YOU ENCOUNTER THE FOLLOWING BARRIERS TO PATIENT INVOLVEMENT? SELECT ALL THAT APPLY.

	Very often	Often	Sometimes	Rarely	Never
Lack of personnel for organising and managing activities related to patient involvement	☐	☐	☐	☐	☐
Lack of financial resources	☐	☐	☐	☐	☐
Regulatory and legal constraints	☐	☐	☐	☐	☐
Patient community is fragmented; there are too many actors and organisations, it is not clear whom to involve	☐	☐	☐	☐	☐
Patient organisations do not collaborate	☐	☐	☐	☐	☐
The political environment should be more supportive of patient involvement	☐	☐	☐	☐	☐
Patients are afraid of the risks in medical research and development	☐	☐	☐	☐	☐
Patients need to understand better their active role related to decisions and actions affecting their health	☐	☐	☐	☐	☐
Patients need more knowledge and skills to engage with their healthcare provider	☐	☐	☐	☐	☐
Patients need more knowledge and skills to engage in medicines research and development	☐	☐	☐	☐	☐
Patients need to be seen as equal partners in medical research and development	☐	☐	☐	☐	☐
There are not enough opportunities for patients to engage in medical research and development	☐	☐	☐	☐	☐
Patients do not have access to relevant information about medical research and development	☐	☐	☐	☐	☐
Individual patients are biased and are not representative of the larger patient community	☐	☐	☐	☐	☐
Patients are reluctant to engage in new roles and activities related to medical research and development	☐	☐	☐	☐	☐

The industry codes (regulatory perception, see pharma.be code) does not support working with the patients	☐	☐	☐	☐	☐
Healthcare providers do not support initiatives on improved patient-physician communication	☐	☐	☐	☐	☐
Patients are not prepared to use new technologies and to follow healthcare digitalisation	☐	☐	☐	☐	☐

Other, please specify:

4. IN YOUR OPINION WHAT ARE THE BEST WAYS TO IMPROVE PATIENT INVOLVEMENT? PLEASE TYPE YOUR ANSWER BELOW.

PLEASE EVALUATE THE RELATIONSHIPS BETWEEN YOUR ORGANISATION AND EACH DIFFERENT TYPE OF ACTORS INVOLVED IN MEDICAL RESEARCH AND DEVELOPMENT ACCORDING TO THE STATEMENTS BELOW. MULTIPLE RESPONSES CAN BE SELECTED. *

5. PLEASE INDICATE FREQUENCY OF COLLABORATION BETWEEN YOU/YOUR ORGANISATION AND DIFFERENT TYPES OF PARTNERS. *

	Very often	Often	Sometimes	Rarely	Never
Industry	☐	☐	☐	☐	☐
Academia	☐	☐	☐	☐	☐
Individual patients	☐	☐	☐	☐	☐
Patient organizations	☐	☐	☐	☐	☐
Policy makers and regulators	☐	☐	☐	☐	☐
Health care professionals	☐	☐	☐	☐	☐
Payers	☐	☐	☐	☐	☐

6. DO YOU UNDERSTAND THE ROLES OF THE FOLLOWING ACTORS IN MEDICAL RESEARCH AND DEVELOPMENT?

	YES	NO	I would like to understand their role better
Industry	☐	☐	☐
Academia	☐	☐	☐
Individual patients	☐	☐	☐
Patient organizations	☐	☐	☐
Policy makers and regulators	☐	☐	☐
Health care professionals	☐	☐	☐
Payers	☐	☐	☐

7. WHAT IS YOUR PERCEPTION ABOUT THE FOLLOWING ACTORS IN TERMS OF PATIENT INVOLVEMENT?

	POSITIVE	NEGATIVE	HARD TO SAY
Industry	☐	☐	☐
Academia	☐	☐	☐
Individual patients	☐	☐	☐
Patient organizations	☐	☐	☐

Policy makers and regulators	☐	☐	☐
Health care professionals	☐	☐	☐
Payers	☐	☐	☐

8. DO YOU TRUST EACH OTHER?

	YES	NO	HARD TO SAY
Industry	☐	☐	☐
Academia	☐	☐	☐
Individual patients	☐	☐	☐
Patient organizations	☐	☐	☐
Policy makers and regulators	☐	☐	☐
Health care professionals	☐	☐	☐
Payers	☐	☐	☐

9. WHICH CHARACTERISES YOU BEST? SELECT ALL THAT APPLY.

☐ I am an individual patient

☐ I am a patient representative

☐ I represent a patient organisation

☐ I work for a patient organisation,but I am not patient myself

☐ I represent a pharmaceutical company or a biotech company

☐ I represent academia

☐ I represent policy makers and/or regulators

☐ I represent health care professionals

☐ I represent industry or professional association

☐ I represent payers

☐ Other, please specify:

10. WHICH THERAPEUTIC AREA/AREAS DO YOU REPRESENT?

☐ Cardiovascular

☐ Diabetes

☐ Oncology

☐ Metabolic diseases

☐ Infectious diseases

☐ Psychiatry

☐ Neurology

☐ Rheumatology

☐ Vaccines

☐ Other, please specify:

11. WHERE ARE YOU/YOUR ORGANISATION BASED?

☐ Flanders

☐ Wallonia

☐ Brussels

☐ Other, please specify:
